# Managing misaligned paternity findings in research including sickle cell disease screening in Kenya: ‘Consulting communities’ to inform policy^☆^

**DOI:** 10.1016/j.socscimed.2013.07.028

**Published:** 2013-11

**Authors:** Vicki Marsh, Francis Kombe, Ray Fitzpatrick, Sassy Molyneux, Michael Parker

**Affiliations:** a Kenya Medical Research Institute (KEMRI) Wellcome Trust Research Programme, Kilifi, Kenya; b Centre for Tropical Medicine, Nuffield Department of Medicine, Oxford University, UK; c The Ethox Centre, Public Health Department, Oxford University, UK; d Public Health Department, Oxford University, UK

**Keywords:** Kenya, Misaligned paternity, Genetic testing, Genetic and genomics research, Community consultation, Empirical ethics, Sickle cell disease, Africa

## Abstract

The management of misaligned paternity findings raises important controversy worldwide. It has mainly, however, been discussed in the context of high-income countries. Genetic and genomics research, with the potential to show misaligned paternity, are becoming increasingly common in Africa. During a genomics study in Kenya, a dilemma arose over testing and sharing information on paternal sickle cell disease status. This dilemma may be paradigmatic of challenges in sharing misaligned paternity findings in many research and health care settings. Using a deliberative approach to community consultation to inform research practice, we explored residents' views on paternal testing and sharing misaligned paternity information. Between December 2009 and November 2010, 63 residents in Kilifi County were engaged in informed deliberative small group discussions, structured to support normative reflection within the groups, with purposive selection to explore diversity. Analysis was based on a modified framework analysis approach, drawing on relevant social science and bioethics literature.

The methods generated in-depth individual and group reflection on morally important issues and uncovered wide diversity in views and values. Fundamental and conflicting values emerged around the importance of family interests and openness, underpinned by disagreement on the moral implications of marital infidelity and withholding truth. Wider consideration of ethical issues emerging in these debates supports locally-held reasoning that paternal sickle cell testing should not be undertaken in this context, in contrast to views that testing should be done with or without the disclosure of misaligned paternity information. The findings highlight the importance of facilitating wider testing of family members of affected children, contingent on the development and implementation of national policies for the management of this inherited disorder. Their richness also illustrates the potential for the approach adopted in this study to strengthen community consultation.

## Highlights

• Community consultation on misaligned paternity in Kenya highlights morally important diversity.
• Openness and family interests are central conflicting values in debates on misaligned paternity.
• In research paternal requests for sickle cell disease testing generate prohibitive moral challenges.
• Informed deliberative group discussions strengthen ethical outcomes of community consultation.

## Introduction

The benefits and harms of sharing incidental findings on misaligned paternity during biomedical activities have been raised as an ethical issue in the literature from many parts of the world (Lucassen & Parker, 2001; Ross, 1996; Turney, 2005; Young et al., 2009). Given the concentration in high-income countries of biomedical activities likely to show this type of genetic information, guidelines, commentaries and empirical research on sharing incidental misaligned paternity findings have largely focused on those settings. The consensus of guidelines is that incidental misaligned paternity information should generally not be shared with parents, albeit with some controversy, reflecting reasoning that genetic testing should not be used in ways that disrupt families (Lucassen & Parker, 2001). In large-scale international surveys of attitudes to disclosing misaligned paternity findings in genetic testing, a majority of professionals expressed this attitude, although many women surveyed in the USA held a different view (Wertz, Fletcher, & Mulvihill, 1990; Wertz & Fletcher, 2004).

Recently, the question of potential for benefits and harms from sharing misaligned paternity information arose during a genomics study at an international biomedical research programme in Kilifi in coastal Kenya. The study, addressing genetic susceptibility and resistance to common serious childhood illnesses, included screening for sickle cell (SC) disease in a population of around 15,000 healthy infants (Marsh, Kamuya, Mlamba, Williams, & Molyneux, 2010). SC disease, a serious genetic disorder, occurs in just under 1% of infants in this area but is not well recognised within the community as a biomedical condition (Marsh, Kamuya, & Molyneux, 2011a). Parents of children found to have SC disease in the genomics study were informed of this result, and referred to a dedicated clinic at the district hospital - run collaboratively between researchers and government health providers – for counselling and long term care (Marsh et al., 2010).

Given the autosomal recessive inheritance of this condition, both parents of an affected child must be carriers of at least one sickle cell gene; a status referred to as having ‘sickle cell trait’. As a corollary, where the social father of an affected child does not have either SC trait or SC disease, he cannot be the biological parent of that child. One unexpected outcome of sharing SC disease information in affected children during the genomics study, reported in detail elsewhere, was the emergence of several requests for paternal testing for SC trait, related to paternal denial of genetic responsibility for their child's condition (Marsh et al., 2011a). Some degree of paternal denial was described as part of a wider cultural tendency for mothers to be seen as mainly responsible for health problems in children in this setting. Paradoxically, this risk of maternal blaming was seen as potentially both *reduced* or *increased* by researchers disclosing information on the genetic roles of parents in SC disease, depending largely on influences within the family and at wider structural levels. Where fathers understood and accepted information on their genetic role in SC disease, paternal denial could be countered. Where explanations were interpreted differently, or not accepted, fathers might continue to deny their role. In others, shared understanding of the inheritance of SC disease might still be associated with paternal denial through doubts about biological fatherhood. Requests for paternal SC carrier testing were seen as particularly likely in families where fathers denied their role in their child's condition. In this way, researchers in Kilifi seemed to be presented with a moral dilemma in deciding how to respond to requests for paternal SC testing.

This paper reports on a study set up to consult a range of residents in Kilifi on the way researchers should respond to requests for paternal SC testing, including whether findings showing misaligned paternity should be shared. The consultation aimed to support the development of local policy on this potentially sensitive issue, as part of a wider research activity to explore residents' perceptions of SC disease and views on sharing information on the condition. Consulting people who will be affected by research policies in this way is widely recognised as morally and practically important, particularly where there may be significant differences between researchers and those who participate in studies, for example, in their technical knowhow, wealth, culture and language (Emanuel, Wendler, Killen, & Grady, 2004). In relation to sharing genetic findings, research ethics guidelines and commentaries highlight the importance of taking account of grounded views on the nature of possible harms and benefits in making decisions about disclosure, including how ethical challenges related to community interests should be met (Knoppers, Joly, Simard, & Durocher, 2006; Ravitsky & Wilfond, 2006).

There are many methodological and theoretical challenges in the literature on undertaking ‘community consultation’ to strengthen ethical practice, including how ‘communities’ are identified and represented (Kamuya, Marsh, Kombe, Geissler, & Molyneux, 2013; Tindana et al., 2007); how views are elicited, particularly around unfamiliar topics (Parker et al., 2009); and how these views should be fairly taken forwards to inform practice, as a normative rather than descriptive process (Dunn, Sheehan, Hope, & Parker, 2012; Ives, 2013). The consultation methods described in this paper draw upon principles of deliberative ethics in which public discussion is seen as central to the identification and analysis of ethical issues, as a substantive and pluralist model (Parker, 2007). Through this description, we make a contribution to the methodological literature on empirical ethics, although it is beyond the scope of the paper to describe the place of this study within current epistemological debates (Dunn et al., 2012; Ives, 2013). Rather, we show that a rich account of informed ethical reflection *by people affected by a specific moral dilemma* can be achieved through qualitative methods based on a structured and deliberative type of dialogue; and that the outputs are highly relevant to an overall process of normative analysis.

## Methods

### Study site

The Kenya Medical Research Institute (KEMRI) Wellcome Trust Research Programme, and the setting of its main centre in Kilifi County on the coast of Kenya, have been described in detail elsewhere (Marsh et al., 2010). In summary, Kilifi County includes rural and semi-urban populations of around 1 million; subsistence farming is the primary livelihood and between 55% and 65% households live below the poverty line (Virtual Kenya, 2011). The study was conducted within the population of 260, 000 people included within the research programme's Health and Demographic Surveillance System (KHDSS) that accounts for around 60% of admissions to the district hospital (Scott et al., 2012). This population constitutes the ‘community’ referenced throughout this paper. The majority of residents are Mijikenda (Parkin, 1991); 47% describe Christianity, 13% Islam and 24% traditional beliefs as their faith system. 45% adults reported an inability to read a newspaper or letter during randomised household surveys in 2005.

### Study population, sampling and data collection

Between December 2009 and November 2010, 63 Kilifi residents in the KHDSS area were engaged in a series of consultation activities to explore their views on the way researchers should respond to requests for paternal SC disease testing and sharing findings showing misaligned paternity. Drawing on experience in community engagement at the research programme over many years (Marsh, Kamuya, Parker, & Molyneux, 2011b; Marsh, Kamuya, Rowa, Gikonyo, & Molyneux, 2008) the consultation was planned as a series of small group discussions (9 groups) each with 3–6 people and held in two stages, and individual interviews (8).

Table 1 gives a summary description of participants. To inform practice, the consultation aimed to take account of the range of views likely to be encountered within the area, and to include those of mothers with an affected child. *A priori* purposive sampling was based on exploring diversity, using criteria of role, gender and rural/urban geographic residence, and all groups included participants of different ages, religion and educational status. Types of residents included: i) those working full time within the research programme (20), including Community Facilitators, Field Workers (front-line staff supporting studies in informed consent processes, interviews and sample-taking), Data Entry Clerks and a Scientist in training; ii) District Health Managers (4); iii) Administrative leaders, Chiefs and Assistant Chiefs (18); iv) KEMRI Community Representatives (KCRs) (18), who are ‘typical’ residents selected by their local communities to support consultation on research-related issues (Kamuya et al., 2013; Marsh et al., 2008); and v) Mothers of affected children (3), not belonging to the above groups. Each data collection activity (interview or group discussion) included participants from only one of the types of residents described.

The discussion method modified that typically used in a focus group discussion (Bryman, 2004) to build in greater involvement by facilitators in directing the discussion and using probes to support individual and group reflection and debate, in keeping with principles of deliberative forms of empirical ethics and similar approaches used by others (Ives, 2008; Ives & Draper, 2009; Parker, 2002). For each group involved in a discussion, two meetings were held one week apart, using local venues (for most participants) or the research centre (for staff members), and the participants' language of choice (English, Kiswahili or local language). Separation into two stages aimed to: i) extend the amount of time for engagement, to support information sharing given the unfamiliarity of many topics; ii) provide greater opportunity for reflection on new understandings between discussions; and iii) allow revisiting of views over time, to strengthen the trustworthiness of data in relation to novel topics. The first stage mainly used participatory processes to share information on SC disease, including its prevalence, health implications, management and inheritance, and the risks of future children being affected where both parents have the trait. The second stage primarily aimed to generate deliberative discussions on the importance of sharing SC disease and SC trait findings in research, using different scenarios for these forms of the condition. In the second stage discussions, facilitators aimed to: explore the views of all participants as far as possible; use non-judgemental probes to explore reasoning and promote reflection, particularly around any emerging morally relevant issues; avoid consensus building; and pay attention to the voices of the most vulnerable within the population, taken here as parents and families of children with SC disease (Ives, 2008; Parker, 2007).

### Data management and analysis

Field notes were made during and immediately after meetings. Discussions were recorded, transcribed and translated into English, including a total of 48.5 h of recordings. Translations were undertaken by note-takers in meetings, experienced staff with fluency in local languages and English, and checked by FK. The study team held debriefings after each discussion, using emerging findings to inform on-going development of the topic guide.

Data were managed using Microsoft Word applications, anonymised through coded identities. Analysis used a modified Framework Analysis approach (Green & Thorogood, 2007), including in-depth reading of transcripts, making detailed summaries of discussions, and development of analysis charts - by individual participant and group - to capture the range and progression of views in each interview or discussion. Charts were structured to systematically describe views on how researchers should respond to requests for paternal SCD testing and manage information on misaligned paternity, including underlying reasoning, strength of opinion, and changes in and influences on views. Charting themes were identified deductively, drawing on topic guides, and inductively, capturing new issues emerging during discussions. Analysis was primarily conducted by VM, with support from other authors, including cross-checking and discussions around coding of data within analysis charts.

Whilst we aimed to maintain high levels of reflexivity in this research, the study design, data collection and analysis of findings have obvious potential for influence from the positions of the researchers involved in this study, including VM, SM and FK having worked in the research programme for more than 15 years. The collaborative nature of this study and the varied backgrounds of the investigators, including in bioethics, social science, public health and community engagement, was constantly drawn upon to support reflexivity.

### Ethical review

The study was approved by the KEMRI Scientific Steering and Ethical Review Committees and the Oxford Tropical Research Ethics Committee (OXTREC) at Oxford University. The manuscript is published with the permission of the Director, KEMRI.

## Findings

Findings are presented as perceived reasons researchers should agree to requests for paternal SC screening, and reasons for and against sharing information with parents on misaligned paternity. Further sections describe emerging underlying values; and perceptions of researchers' responsibilities in relation to balancing these values.

### Reasons for agreeing to test fathers' sickle cell status: countering paternal denial

Where fathers of children shown to have SC disease requested testing of their own SC status, many residents, including all mothers with an affected child, thought this should be done. Their reasoning was that such fathers were likely to have been prompted by doubts about paternity and – since it was assumed this doubt would usually be unfounded – parents and children would benefit from confirmation of the father's SC carrier status:

“There, it's important for all fathers to be tested for it to be known, because those who get children outside their marriage are very few. You might get for example in a group of twenty people, maybe it's only one person who went out of the marriage, and shouldn't the others be told?” (Mot/P1)

This view was particularly underlined by perceptions that many fathers would have concerns about their paternity in this situation, and that continued uncertainty would have serious consequences for the child, the mother and the stability of the family. Increasing risks of separation were perceived where more than one affected child was born. Since the risk of fathers *not* being shown to be carriers was considered low, potential harms from showing misaligned paternity would be infrequent. In addition, many residents felt that mothers implicated by a misaligned paternity result would have already compromised her position and their family's stability:

“I also agree that the test should be done… it means their relationship is not good…and it will assist in other ways, like when this lady is not faithful, then she can bring other types of illnesses in the family... maybe she will get AIDS and bring this to the family” (IDI07/P1, female).

In this way, sexual faithfulness in marriage was sometimes seen as an intrinsic moral value, particularly for women, but its importance was often described in protecting families from HIV/AIDS and other sexually transmitted infections (STIs). Others felt that information about the father's SC status could be withheld if he was shown not to be a carrier, justified by a positive motivation to support the family's and child's interests:

“It [lying] will be fine because lying to someone is not cutting them. Lying, you know there is a kind of lie you can protect with, so that you get something to help, and another kind where if you follow it, you will go to hell, now this one is not that of hell (all laughing), this one is for saving someone!” (KCR03/P4, male) The idea of lying was also often ‘softened’ in different ways, for example, as “something to add on top of that [truth].” (Chiefs 01/P6, male)

### Reasons not to share misaligned paternity information: risks of harm to children, mothers and families

As suggested above, although there were clear reasons for agreeing to test fathers and share information on their SC status, there was also widespread concern across all groups that sharing misaligned paternity information had a high risk of harm for implicated mothers, affected children and families. Mothers whose partners were already suspicious about paternity were seen as particularly vulnerable. Residents described very different potential consequences of sharing misaligned paternity information, depending on family- and wider-level influences, discussed below. At a minimum, some disruption to the family was anticipated, for example “there will be no peace in that house” (KCR02/P4, male). But all agreed that harms could include family separation, an outcome seen as potentially causing very serious hardship to mothers and children who – in this traditionally patrilineal culture (Shaw, 2006) – were likely to be ‘sent back’ to the maternal clan, where social and financial support might not be available:

“Definitely if the results are out and the man is not a carrier, and if we give out the results, that home is broken already.”(Chiefs 02/P1, male)

The affected child's chronic ill health was seen to increase risks of family separation:

“…he [social father] won't agree to live with a sick child, he doesn't know where it came from, he won't also agree and that's why he will have to tell you, okay, where you got this one from, okay, take him back.” (Mot/P2)

Even if the mother were not sent away, she and her child could still be adversely affected:

“I think some might agree for you to stay there but he [affected child] won't be given the same care as the others...because he [father] will obviously know that this child is not mine” (Mot/P1).

At the extreme end of reported harms, some residents perceived risks of serious physical assault to the mother. A community facilitator described misaligned paternity as a “*panga* (machete) case”. Several residents in one group (KCR03) insisted that misaligned paternity findings could not be disclosed, given risks the mother might be fatally assaulted. In this group, the prominence of jealousy as a feature of some marriages was linked to “little chance of forgiveness” (KCR03/P3). These very serious harms might also affect a suspected biological father, as suggested by a health manager:

“It is a very big worry… if it does not prove…that both of them are carriers, then it would probably lead to breakages of families, yeah, and even witch hunting, because…they would want to know who the father to this child is.” (IDI13, male)

### Reasons to test father and share misaligned paternity findings: harms may not be serious

Variation in the nature and likelihood of perceived harms from sharing misaligned paternity findings were explained on the basis of two key features; the possibility of forgiveness in the family and the ability of mothers to manage independent lives if separation occurred.

#### Forgiveness as a moderator of harm

Many residents saw the nature of different relationships, especially trust, as key in fathers' request for SC testing, and responses to misaligned paternity findings:

“There are so many reasons which can cause that [breakup]. So if it is a family…[where]…the relationship is that rich and strong, I think …even if the man learns that this could not be his child…they might discuss and resolve.” (IDI07/P3, female)

“The child was … found to have sickle cell…it was explained very well to us by the doctor. Even these issues concerning… going out [marital infidelity] were also explained to us…but if you do not have trust in the house, this issue will come up, because of lack of trust.” (KCR02/P2, female)

There was considerable variation in residents' views of the possibility of forgiveness in this situation. Direct examples of strongly nonjudgmental attitudes included a comment that “it's normal for people to be lost” (KCR03/P5) and a local saying that “*kufanya kosa si kosa, na kuregelea, kosa ndio kosa*” (Kiswahili) (KCR02/P2), meaning ‘a mistake is only a mistake when it's repeated.’

Some health managers and chiefs gave an indication that unfaithfulness in marriage might have been more acceptable in the past:

“…of course nowadays things are changing, but there was the, not really a leeway, but I think it was like very obvious when a woman moved out with other men, and it was also very normal for a man to move out with other woman… that's why the man can [easily] blame the woman...because to them that thing has been happening.” (IDI14, female)

Similarly, a chief argued that ‘traditionally’ the importance of a new child in a family would outweigh other considerations of jealousy or biological paternity; some described older men as more likely to accept a child conceived ‘outside marriage’. In some cases, the wider family might be drawn into resolving marital disputes, including those around biological paternity. The maternal family might provide mothers with a temporary home at times of conflict, while actively seeking to resolve underlying issues. Separation was said to be “not just a case of what the parents want to do”. Many residents also described an influence from Christian beliefs in ideals of forgiveness. Although the child's chronic ill health and awareness of the link between sexual infidelity and HIV/AIDS may counter these non-judgemental attitudes, these views also suggest the concept of fatherhood often encompasses a wider social rather than a purely biological meaning, as described in the UK and Australia (Ives, Draper, Pattison, & Williams, 2008; Turney, 2005).

#### Mothers' resources and social networks

In cases of family separation, the mother's social and economic resources were described as important determinants of the consequences for the mother and child. In particular, relatively well-educated and employed female community representatives tended to express positive views about many mothers' ability to look after their own interests. A resident from a rural area with only partial primary education also asserted her independence:

“If I see my partner has become angry such that I suspect he might beat me or say some other words, I won't keep quiet, I will go anywhere to look for assistance… there are ways of sorting out these things in our homes, and so we shouldn't be hiding anything...” (KCR2/P5, female)

### Emerging values and their prioritisation

In this section, we turn to describing two important underlying sets of values that underpinned the reasoning described above. The predominant value was the importance of protecting vulnerable children, their mothers and families, described here as ‘family interests’. A second, and often conflicting, value was ‘openness’ or truth, often linked to protecting trust in researcher–participant relations and societal issues of public health and development.

#### Family interests

‘Family interests’ is used here to represent a value with perceived intrinsic and instrumental importance. Intrinsically, the family unit – or ‘group of family members…closely related by living arrangement or by commitment’ (Lindemann Nelson & Lindemann Nelson, 1995) (p6) – was spoken of as representing a harmonious life for individual members and the wider community. Instrumentally, the interests of individuals were to varying degrees protected and nurtured by family membership. From both perspectives, the interests of individuals were generally seen as aligned with that of the family, with some controversy over balancing fathers' and family interests. Concealing the truth about a father's (negative) sickle cell trait status was seen by some as acting against his interests either by infringing his right to knowledge or by the unfairness of being ‘tricked’ into financial support for children that were not his. In contrast, other community members, including men, articulated a right not to know about misaligned paternity, given the threat to family interests:

“If he tells me the truth that will be very good, but things will go crazy when we go home, and … I'm sure my partner won't stay at home, she will go. But because there's that will to keep the family together… even though we are not saying it should be like that, let him lie to me so that I continue to stay with my wife.” (KCR03/P3, male)

Distinctions between the importance of a father's rights to knowledge and protecting family stability were often unclear but greater importance was generally attached to the latter, for example, drawing on comparisons with national HIV/AIDS policy for discordant couple counselling:

“I would borrow heavily from the VCT counselling protocol, that if telling the results to your wife…will lead to either separation or probably cutting down some of the support that used to direct to such a person, then I would better not tell you the results.” (IDI 13, male)

#### The importance of truth/openness

Many arguments were underpinned by values of truth telling and openness. The strongest argument made in support of openness, and against the concealment of misaligned paternity findings, was the risk of loss of trust through later discovery of hidden information:

“Haven't you seen a person going from one hospital to the other to be tested? He is not sure…[about his positive results so decides]… ‘let me seek advice from other people’...So I think it's important for him to be told” (Mot/P3)

Some participants in nearly all groups saw loss of trust as more harmful, particularly in the long term, than risks associated with sharing misaligned paternity information. The problem described by a chief as people later ‘looking at you with a different face’, was seen by some staff as particularly important for researchers, with impacts of loss of trust at individual, family and wider community levels.

A second argument for openness, made by some members in all community groups, was that concealment could support continued sexual unfaithfulness, leading to higher risks of HIV/AIDS and other sexually transmitted infections. Conversely, being open about misaligned paternity findings could encourage greater faithfulness:

“Eeh, now you, it shows this child is not mine, maybe I can chase her to go to her home and then when she reaches there, the neighbours may say ‘why is mother so and so at his home?.. aah she was chased, her husband said the child is not his because of this and this’. Now the other women will learn from her!” (laughing) (KCR1/P3, female)

These more judgemental attitudes towards unfaithfulness stand in contrast to ‘traditional’ attitudes of acceptance and forgiveness described earlier. Relatedly, a risk to trust was described where researchers were perceived as being complicit in encouraging unfaithfulness through concealment of information.

A third way in which some residents argued in support of openness around misaligned paternity findings was in empowering family members through knowledge, seen as intrinsically important as well as important for longer term individual and societal benefits:

“If we say that other things should be hidden, will be kept under the mattress, that will be…meaningless, and so the doctor should explain, and the father should be tested if he requests, and the mother should be tested too if she requests. There should be freedom.” (KCR2/P5, female)

Across these discussions, questions about the advisability of paternal testing and sharing misaligned paternity results in SC disease often prompted reflection on HIV/AIDS control policies, specifically referencing Voluntary Counselling and Testing (VCT) and discordant couple counselling as measures potentially applicable to SC disease. Most residents were very positive about the HIV/AIDS control activities; openness in testing and disclosing HIV status were seen as instrumental in reducing stigmatisation of that disorder:

“…there is VCT [for HIV] going on at the moment, and everywhere people have been singing about it, that both the father and the mother should go for testing...Also in that [SC disease] situation… the doctor will explain to you, and if you understand, like the way the HIV/AIDS doctors or counselors explain to us... you will continue living. Let it be like that situation, so as for us not to be confused, to avoid shutting down the child's future” (KCR2/P5, female)

In promoting openness towards public health gains, an important misconception for some was that SC disease would - like HIV/AIDS - show epidemic features at a population level:

“This condition [SC disease]…because people were not informed, carriers kept on marrying each other, now there are more people who are born positive…So if the test is done and … they become fully aware…this will discourage that situation where carriers marry each other, more and more, more and more.” (IDI07/P3, male)

In any case, openness in current HIV/AIDS policy was not universally persuasive when applied to SC disease. Notably, the group of community representatives who perceived greatest risks of serious harm to mothers through showing misaligned paternity did not consider ‘openness’ a good way forwards.

### Researchers' responsibilities

Against this background of conflicting views and values, perceptions of how researchers should handle information sharing on misaligned paternity were strongly influenced by the assessment made of foreseeable benefits and harms of disclosure. Many residents believed that researchers should protect the interests of children with SC disease and their mothers by withholding misaligned paternity findings, but with varying degrees of conviction:

“… you doctors should sit, because you know if you say the truth, you will have caused a fight in the home, so you should look for a way of hiding the truth so that at least there be peace at home, and continue caring for the child.” (KCR03/P6, male)

Most saw it as important that, at least, researchers/doctors should not directly raise questions about misaligned paternity (for example, by offering parental testing) and, in explaining the genetic roles of parents, try to avoid generating requests for parental testing. In strong contrast, some saw misaligned paternity as a domestic issue:

“ Let him be tested...you have done your part, the results that come out belong to them...your part is to educate him...the results are not your problem, now that's for him and his family, they will decide on their own the way they will live…you will have done your duty.” (KCR1/P2, female)

## Discussion

This study was set up to examine the views of a wide range of Kilifi residents on the way researchers should respond to paternal requests for SCD screening, including handling findings showing misaligned paternity, in the context of a genomics study on infant susceptibility and resistance to common causes of serious morbidity in Africa. The data collection methods were based on participatory information sharing and structured deliberative discussion, to facilitate informed reasoning and reflection on ethically important features of the debate. An in-depth examination of this relatively novel method of consultation, and its place within debates on the value of empirical approaches to ethics (Dunn et al., 2012; Ives, 2013) is beyond the scope of the current paper. Nevertheless, the findings show that rich and complex accounts of individual views can be explored in this way, and that ethically important forms of diversity emerge, with interrelated individual-level and contextual influences (Horstman & Finkler, 2011). Given the overall aims of this study to consult a ‘community’ of local residents on research policy, our main aim in this discussion is to draw together the diverse views of a range of residents and a wider bioethics literature to support ethical reflection on what would represent a locally responsive and widely justifiable approach to working with misaligned paternity findings in research in this and other similar contexts.

### Taking forwards diverging views and values in community consultation

Researchers were seen to have three options in responding to requests for paternal SC screening: refusing to test; agreeing to test and sharing all results, including those indicating misaligned paternity; and agreeing to test and sharing true paternity outcomes but withholding misaligned paternity results. The fundamental unresolved judgements between these positions concerned:

i) Researchers' responsibilities to avoid deception and maintain trust *versus* those to protect family interests where misaligned paternity is shown.
ii) The degree of acceptability *versus* unacceptability of sexual unfaithfulness in marriage, such that where misaligned paternity is shown, the interests of implicated mothers and families could be reasonably infringed.

Two perceptions key to these arguments were based on insufficient understanding at some level. The first was a misconception about the nature of SC disease, which was seen as a dangerous new epidemic with a potential for rapid spread. This view gave weight to the importance of public health interests, including over those of some individuals, to ‘control’ the disorder. Greater awareness of the true nature of this genetic condition would be important to rebalance this assessment. The second important and contested perception concerned the likely prevalence of misaligned paternity findings amongst children in this community, for which no evidence currently exists.

One approach to taking forwards the unresolved tension in residents' views is through reflection using a wider biomedical ethics literature, without losing focus of important and widely shared values in residents' debates. There are limitations to drawing on the literature, since most guidelines and commentaries on disclosing misaligned paternity concern incidental findings, and relationships with adult participants/clients. This differs from requests for paternal testing and studies involving young children, as was the case in the Kilifi genomics study. However, ethical analyses of fathers' rights to paternity testing in relation to compensation claims in the UK have underlined similar values to those emerging in this consultation, including a fundamental importance of the interests of children, and relationships between children and their parents (Draper, 2007; Draper & Ives, 2009). Commercial paternity testing offers some comparisons (Davis, 2007) but here there are arguably even greater differences in the way that responsibilities might be understood (Richardson & Belsky, 2004).

Lucassen and Parker (2001) review of guidelines on sharing incidental misaligned paternity findings reports a consensus against disclosure, in health research or care, based on breaching the confidentiality of mothers; dissuading others from testing; and the pressure that misaligned paternity knowledge puts on couples, including the possibility of violence and breakup (Lucassen & Parker, 2001). Obligations of non-disclosure are based then on duties not to harm. In contrast, reasons for disclosure are: avoiding deception and associated loss of trust; ensuring that accurately informed choices can be made in future; and challenges to the autonomy of the parents, leading to charges of undue paternalism on the part of the doctor/researcher, if this information is withheld (Lucassen & Parker, 2001; Ross, 1996). These arguments are well reflected in the controversy in Kilifi. Of particular relevance are concerns that sharing information may generate conflicts, violence and family breakup; and that non-disclosure involves deception, and may lead to loss of trust between participants and researchers in future.

### Is it reasonable to withhold negative SC trait results?

In Kilifi, positions for and against withholding negative SC trait findings in fathers of affected children were primarily linked to perceived consequences. Fathers' rights to knowledge about their SC status, for example, were not generally seen as more important than the interests of the family he is part of. Although recognised as a wrong, false reassurance to the father about his paternity status was justified by the greater weight accorded to the need to protect the interests of the child and family. However, a further consequence of withholding misaligned paternity findings is generating false anxiety about future reproductive risks (Lucassen & Parker, 2001); a risk not fully considered in these debates. At the same time, discussions had highlighted the serious implications of lifelong anxiety about the health of all future children for parents of a child with SC disease, including family instability. Further, a risk of loss of trust in researcher-participant relationships if findings are withheld and later found out, described by many participants, represents both an intrinsic and instrumental form of harm, with important implications for relationships with the wider community and future research initiatives. Taken together, these arguments strongly suggest that an approach of screening fathers on request but withholding misaligned paternity findings would be difficult to support based on values seen as important by residents in this consultation.

### How can the interests of ‘many faithful’ and ‘few unfaithful’ mothers be balanced?

If misaligned paternity findings should not be withheld, researchers have two possible options; to agree to test fathers and share misaligned paternity results if found, or to refuse to test fathers. As described earlier, the key judgement underpinning views about these options concerned the relative acceptability or unacceptability of sexual unfaithfulness in marriage, and the extent to which this behaviour might make it reasonable to infringe family interests in some cases. All mothers of affected children felt that, where true paternity could be shown, family interests were so important that it would be reasonable to risk adverse consequences in others; a view supported by concerns about risks from STIs such as HIV/AIDS.

Negative attitudes towards misaligned paternity in Kilifi often differed from those described elsewhere in the literature as “moralistic, judgmental, and politicized” (Turney, 2005, p245). Instead, support for sharing misaligned paternity findings was more often based on assessing the consequences for all concerned, importantly including the public health benefits of discouraging marital infidelity, and assuming that misaligned paternity would not be common. This is a challenging assessment to make, particularly where very severe harms, including death, are a possible if relatively unlikely outcome. In general, there is little empirical evidence on the outcomes of disclosing incidental misaligned paternity to couples (Lucassen & Parker, 2001; Ross, 1996; Young et al., 2009), and patchy evidence on misaligned paternity rates in different settings (Turney, 2005; Young et al., 2009). It seems reasonable to accept community views that disclosure could lead to severe harms *in some cases*; a situation where a safety-first principle, proposed for assessing risks of ‘worst-case scenarios’ in genomics research, would be very relevant (Hoedemaekers, Gordijn, Hekster, & van Agt, 2006). Taking these issues into account, a general policy of sharing misaligned paternity information does not seem to be based on a convincing argument of a wide form of positive balance between benefit and harm.

Further reasons – raised but not fully debated in the groups – make it difficult to support a view that misaligned paternity findings provide a reasonable basis for undermining the interests of implicated mothers. Firstly, a single finding of misaligned paternity may not match up well with sexual behaviour or infection risk in general. This feature is particularly important because the main perceived problem with ‘unfaithfulness’ was an increased risk of HIV/AIDS, leading to a form of wrongful discrimination.

Secondly, the consequences of discriminating in this way against certain mothers would almost certainly adversely affect vulnerable children who could not be held responsible for their parents' actions. Draper and Ives' (2009) ethical analysis of the rights of men to paternity testing in the UK similarly concludes that such requests are difficult to support, including arguments for the central nature of the interests of children involved in such disputes (Draper & Ives, 2009). Their analysis points to the importance of making a distinction between social and biological concepts of paternity, recognising the rights and responsibilities (of fathers and their children) as bound up within existing fatherhood relationships, irrespective of genetic links. This reasoning strengthens residents' arguments in this study that researchers should not agree to requests for paternal SC testing, particularly since children with SC disease generally develop symptoms at an age when social bonds of paternity are often already established.

Thirdly, unlike risks of HIV/AIDS, this form of discrimination is inevitably uneven from a gender perspective; acts of sexual infidelity in men carry this biological risk of blame for the women they are involved with, but not for themselves. Gendered blame was a key concern throughout these debates. Finally, there seem to be important issues with seeing ‘paternity testing’ as a responsibility of researchers, and arguably even of providers, with some community members seeing paternity as a private family issue. It is possible to draw on this view – that it is not researchers' responsibility *to protect families from knowledge of misaligned paternity* – to argue that it is also not easy to see a responsibility for researchers to *demonstrate* such a situation.

## Conclusions

Community consultation in Kilifi on the way researchers should respond to paternal requests for SC testing during studies has highlighted residents' main concerns, strong diversity in views, multiple and inter-related individual-level and contextual influences, and the nature of moral tensions associated with assessments of good practice. Although no clear position emerged from the discussions it is possible to propose a conclusion from this consultation through ethical reflection on the implications of views and drawing on the literature, with a particular focus on values seen as important by residents. These findings underline the contribution made by consultation methods including allowing significant two-way information sharing, encouraging in-depth deliberative discussion, and responding to diversity. To inform policy more substantially, these conclusions – and their bases – should be fed back through an iterative process involving community and other stakeholders implicated by a decision (Abma, Molewijk, & Widdershoven, 2009).

From the process used here, we conclude it is difficult to support an argument that researchers should test fathers for SC trait on request since practices of sharing all findings or withholding misaligned paternity findings both conflict with important local and more widely held values. These include the importance of maintaining trust in researcher-participant relationships, avoiding wrongful and gendered discrimination, and supporting the interests of vulnerable children and their families in general, including not creating false anxiety about affected parents' future reproductive risks.

This conclusion is strongly influenced by the absence of national policy on SC disease management in Kenya and low public awareness of the biomedical nature of this condition, all of which suggest that the long term resources needed to limit serious harms associated with paternal testing could not be realistically provided through research funding (Sharp & Foster, 2006). On this basis, a responsibility can be argued for researchers – where studies include testing for SC disease – to promote the development of such services within government health facilities. In future, based on a better understanding of misaligned paternity rates in the community and a national policy on SC disease management supporting provision of health services, family counselling and public information, there are clearly important reasons to facilitate wider testing of family members of affected children. While taking into account challenges in drawing comparisons between SC disease and HIV/AIDS, approaches to couple counselling in control programmes for the latter may act as an important model for disclosing SC trait findings in parents of affected children in low-to-middle income countries in future.

## Figures and Tables

**Table 1:** Summary information for participants.

Role	Total number	Gender M:F	Education (years) Range and median	Religion^a^	SCD history
Staff: Community facilitators	5	4:1	12–16y 14y	4C/1M	No direct history
Staff: Field workers	12	10:2	12–14y 12y	10C/2M	1 – sister has 2 children with SCD 1 – 2 affected children, 2 further children died SCD
Staff: Others^b^	3	0:3	12–16y	3C	1 data entry clerk – carrier, 2 brothers with SCD, 1 died; carrier child
Health managers	4	3:1	16–18y 16y	4C	No direct history
Chiefs/assistant chiefs	18	16:2	7–14y 12y	17C/1M	No direct history
KCRs: 5 chair/vice chairs; 4 secretary/vice secretaries; 9 members	18	9:9	3–16y 8y	14C/4M	1 KCR rural area – 1 child with SCD
Community members: affected mothers	3	0:3	6–12y 6y	3C	2 with 2 affected children; 1 with 1 affected child

a C = Christian; M = Muslim.

b Two data entry clerks and one junior scientist.
